# A natural coastal blowhole as a novel wave energy extraction mechanism; experimental, cfd, and probabilistic evaluation

**DOI:** 10.1038/s41598-026-50798-3

**Published:** 2026-05-07

**Authors:** Taha Rezaei, Akbar Javadi

**Affiliations:** Department of Engineering, University of Exeter, Exeter, EX4 4QF UK

**Keywords:** Wave energy, Natural blowholes, Nature-based solutions model, Chabahar, Monte Carlo framework, Climate sciences, Environmental sciences, Ocean sciences

## Abstract

Natural coastal blowholes are rare geomorphological features in which incident waves induce oscillatory motion within coastal cavities, compressing and venting air through a surface outlet and forming a naturally integrated analogue of an oscillating water column. This study evaluates natural blowholes as a nature-based solution for low-impact wave-energy extraction using a combined experimental-numerical-probabilistic framework, with Chabahar Bay in the Oman Sea as a case study. A laboratory flume model was constructed to reproduce blowhole airflow dynamics, and outlet air velocity measurements were used to compute mass flow rate and validate a CFD model under consistent boundary conditions. The validated CFD framework was then extended to the field geometry to estimate pneumatic kinetic power, yielding a peak outlet velocity of approximately 21.6 m s⁻¹, a mass flow rate of approximately 13.0 kg s⁻¹, and an associated kinetic power of about 3 kW per vent. To represent variability in ocean forcing, a Monte Carlo framework comprising 5,000 realizations was parameterized using regional wave-climate statistics and propagated through the deep-water wave-power formulation to quantify uncertainty in wave-power flux. The mean wave-power flux is 8.7 kW m⁻¹ with a 95% confidence interval of 1.6–22.5 kW m⁻¹, and pronounced seasonal variability is observed, with mean flux ranging from approximately 4.5 kW m⁻¹ in winter to 13.2 kW m⁻¹ in summer. These results demonstrate that natural blowholes can provide measurable, site-specific energy yield while minimizing additional coastal infrastructure, and that probabilistic assessment is essential for quantifying uncertainty and seasonality governing long-term reliability.

## Introduction

The global ambition of achieving a carbon-neutral world by 2050, endorsed by the United Nations, has prompted unprecedented commitments to reduce greenhouse gas emissions^1^. Industrialized nations, responsible for more than 70% of global energy consumption and approximately 65% of total emissions, have pledged to reach net-zero carbon emissions by mid-century^2^. Within this context, ocean energy emerges as a critical, yet underexploited, renewable resource, particularly in coastal regions where demand and opportunity converge^3^. Unlike solar and wind power, which are often limited by temporal intermittency and geographical constraints, wave energy offers greater stability. Moreover, its energy flux is up to 20 times higher than that of other renewable sources^4,5^ positioning it as one of the most promising contributors to future energy systems. Global assessments estimate wave energy potential at around 32,000 TWh per year, of which 2.11 TW (approximately 70%) is technically extractable along coastlines where population density and infrastructure proximity enhance feasibility^2,6^, owing to the proximity of significant global populations to these areas. Notably, about 40% of the world’s population lives within 100 km of coastlines, facilitating the establishment of efficient energy transmission and distribution infrastructures^7^. However, the development of ocean energy systems can introduce environmental and socio-economic pressures, particularly in sensitive coastal zones^8^. This alignment of natural resource and human settlement underscores wave energy’s potential to narrow the persistent supply-demand gap. However, the development of large-scale offshore wave energy farms has also raised ecological and social concerns. Marine habitats may be disrupted, altering the behaviour of key species^9,10^ while the visual impact, interference with fishing grounds, and potential disruption to tourism pose socio-economic challenges^11,12^. Consequently, there is growing recognition of the need to frame energy systems as nature-based solutions (NBS) that integrate with natural processes rather than imposing upon them. This motivates investigation of naturally occurring features, such as blowholes, that inherently integrate with coastal systems. In this regard, natural blowholes geological formations where wave-induced oscillations drive airflow through coastal cavities represent an inherently low-impact alternative. By adapting and enhancing such naturally occurring features, it may be possible to harness wave energy in ways that reduce ecological footprints while contributing to sustainable coastal development^13–15^.

Despite extensive research on oscillating water column (OWC) devices and offshore wave energy farms, natural blowholes have rarely been examined as a systematic energy-harvesting solution. Previous studies have primarily focused on theoretical energy assessments^16^ or offshore installations^17^, leaving a gap in understanding how coastal geological formations can function as nature-based solutions for renewable energy. Unlike conventional oscillating water column (OWC) studies, which primarily focus on engineered devices with tunable geometries and deterministic performance metrics, this study examines a naturally occurring geomorphological conduit whose geometry and boundary conditions are fixed by coastal processes. As a result, the scientific objective shifts from device optimization to validated characterization of airflow dynamics and uncertainty-aware assessment of energy availability^18–20^. The novelty of this work lies in its integrated framework combining laboratory reproduction of blowhole airflow, CFD validation against measured outlet velocity and mass flow rate, field-scale extrapolation to a real natural geometry, and probabilistic quantification of wave-energy availability using a Monte Carlo approach. This integration enables explicit representation of uncertainty and seasonal variability that is not addressed in prior deterministic assessments of blowholes or OWC devices.

## Study area & case study

### Case study

**Fig. 1:**
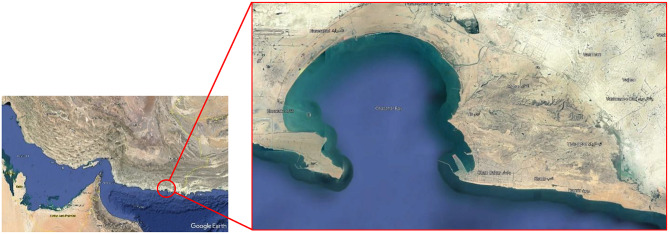
Geographic location of the study area in Chabahar Bay, Oman Sea.

Chabahar Bay, located along the northern coast of the Oman Sea, presents an ideal environment for examining blowhole dynamics due to its exposure to long-period swell and seasonally modulated monsoonal waves. Natural blowholes are rare geomorphological features formed when wave-driven pressure pulses force water and air through confined vertical or inclined shafts. Despite their scarcity, they represent an overlooked mechanism for wave-energy concentration, particularly in regions where steep coastal cliffs interact with energetic wave climates. The study area combines accessible field conditions with a wave regime energetic enough to sustain blowhole activity, providing a suitable testing ground for experimental, numerical, and probabilistic assessments. Chabahar is a city and free-trade zone with an area of 17,155 km^2^ in the southeast of Iran and the north of the Indian Ocean (Fig. 1). The name ‘Chabahar’ derived from the Persian words for ‘four springs’ refers to its mild climate, characterised by relatively temperate conditions throughout the year. It is located at the geographical coordinates of 25.29 degrees north and 60.63 degrees east^21^. The city has a population of approximately 130,000 people and consumes an average of 97 MW average demand. Chabahar has the greatest potential for extracting energy from waves among other coastal cities of Iran. With approximately 265 km of coastline and an estimated average wave-power potential of 5.8 kW m⁻¹, the region has been estimated to support up to 1539 MW of theoretical wave-energy capacity^22^. Chabahar Bay is a natural omega-shaped bay where the ocean climate and wave dynamics are complex and exhibit strong seasonal variability, influenced both by the Shamal winds originating from the Persian Gulf and by the monsoonal systems of the northern Indian Ocean^23^.

One of the key factors in the use of wave energy in this area is the temporal variation of waves power. Due to geographical conditions, Chabahar has hot summers and extremely humid winters, which increases the energy demand for cooling systems. According to Table 1, the highest power of waves in this area is from June to August^22^.

**Table 1:** Monthly wave-power potential along the Chabahar coastline based on regional wave-climate assessments^22^

Month	Wave Power (total MW potential)
January	11.19
February	18.30
March	12.88
April	9.55
May	9.41
June	14.35
July	18.17
August	20.61
September	10.74
October	6.08
November	7.86
December	2.08

## Natural Blowholes

**Fig. 2:**
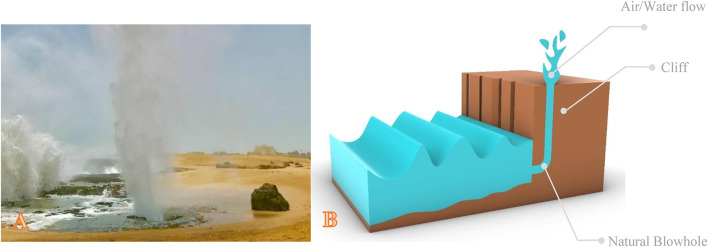
(**A**) Schematic representation of a natural blowhole structure, illustrating the submerged cavity, conduit, and surface outlet. (**B**) Photograph of the natural blowhole in Chabahar, showing recurrent jet formation driven by wave-induced cavity pressurisation^27^.

Natural blowholes are coastal cavities connected to the sea through submerged conduits where wave-driven pressure oscillations force air and water through vertical or inclined outlets. Their formation is typically associated with structural weaknesses in cliffs, fault-controlled fractures, or karstic voids. When incident waves compress water within the cavity, the resulting pressure pulse expels air through the outlet with substantial velocity, operating similarly to an OWC system but without engineered structures (Fig. 2A)^24^. Most of these holes are created along fault lines or islands^25^. In some areas, due to the erosion of the cliffs, such holes are visible^26^. This high-pressure movement of air, which is accompanied by spraying water outside, has a high potential for providing energy using a turbine.

Investigating these natural events offers a significant opportunity to advance our understanding of nature’s ability to generate energy. Understanding the structure and physics of these vents is critical to harnessing ocean energy, especially along coastal areas. Also, identifying these phenomena in different parts of the world and finding beaches that have this potential will be of significant help. Furthermore, implementing these natural phenomena in controlled laboratory environments can provide deeper insight. Such simulations enable a more comprehensive analysis, which is useful in developing accurate and appropriate models of these phenomena.

Owing to their rarity and site-specific geometry, natural blowholes have received limited quantitative analysis. The Chabahar blowhole represents one such site where steep coastal cliffs and energetic monsoon-modulated wave climates combine to sustain recurrent jet events (Fig. 2B). This phenomenon is part of the geomorphological features of the area, which can throw water up to a height up to 10 m^22^. In this study, efforts have been made to implement the physical conditions related to this natural phenomenon in a controlled laboratory environment. Fluent software has also been used to simulate this natural phenomenon. This approach provides the possibility to investigate and analyse the Chabahar blowhole and similar phenomena.

## Methodology

To systematically assess natural coastal blowholes as wave-energy resources, this study adopts an integrated methodology that combines physical experimentation, numerical simulation, and probabilistic analysis. The approach is designed to capture both the deterministic airflow dynamics governing blowhole behaviour and the inherent uncertainty associated with variable ocean forcing. Rather than optimising an engineered device, the methodology focuses on validated characterisation of a fixed geomorphological system and on quantifying the reliability of its energy potential under realistic and seasonally varying wave conditions.

**Fig. 3:**
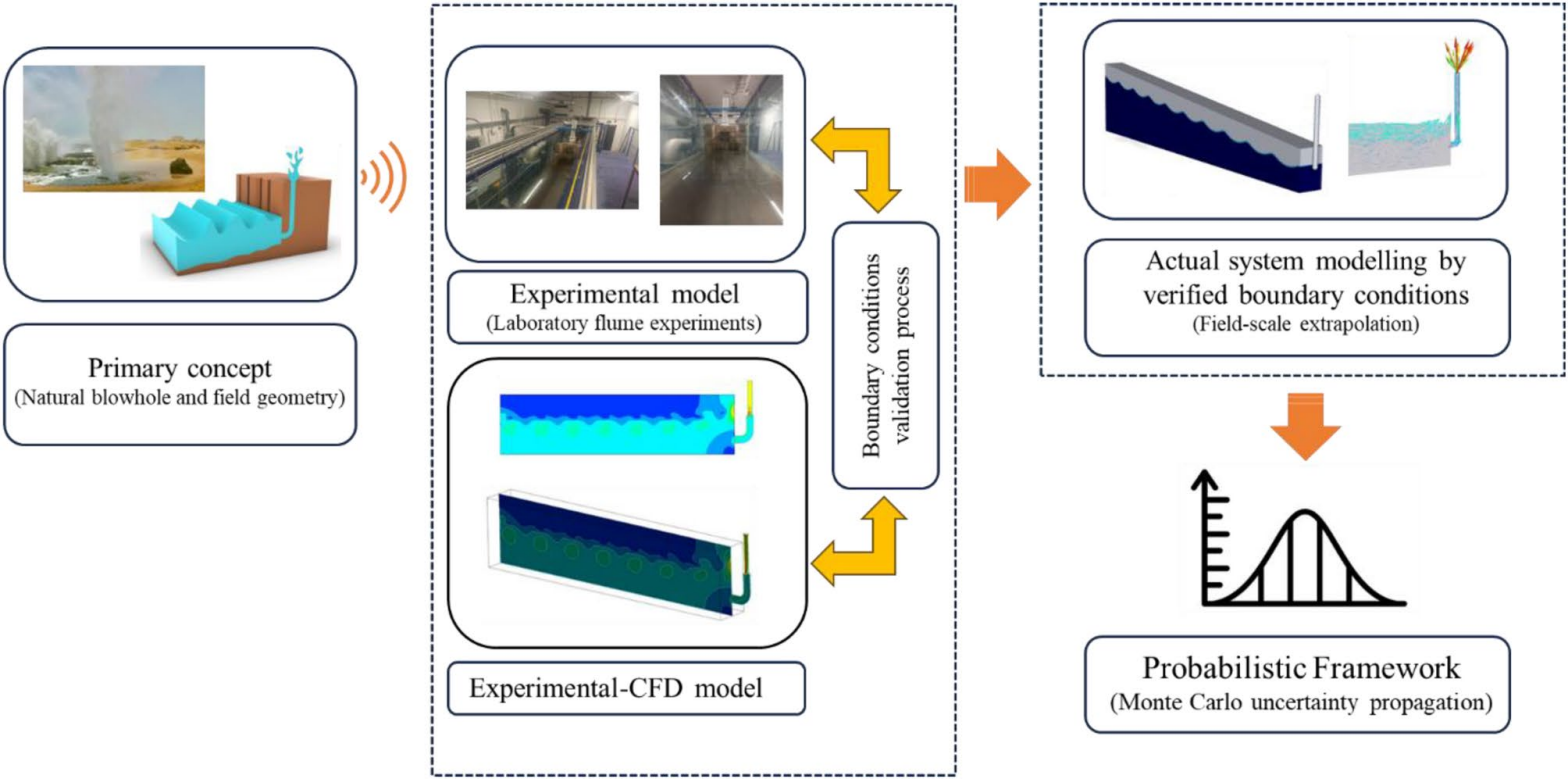
Integrated experimental-numerical-probabilistic workflow for assessing wave-energy potential of natural coastal blowholes, linking laboratory experiments, CFD validation, field-scale extrapolation, and Monte Carlo-based uncertainty analysis.

As illustrated in Fig. 3, the workflow begins with characterization of the natural blowhole geometry and field conditions, followed by laboratory-scale flume experiments that reproduce the essential airflow dynamics under controlled wave forcing. These experimental measurements are used to validate a two-phase CFD model, forming an experimental-numerical feedback loop that ensures physical consistency. The validated numerical framework is then extended to field-scale conditions and coupled with a Monte Carlo approach to propagate uncertainty in offshore wave climate into probabilistic estimates of wave-energy availability.

### Wave power fundamentals

Ocean surface wind-generated waves contain significant energy, derived from the combined vertical and horizontal motion of water particles, which can be converted into electricity^28^. Various technologies have been developed to harness this potential, including point absorbers, oscillating water columns (OWCs), and surface attenuators. Among renewable sources, wave energy is particularly attractive due to its high energy density, which is substantially greater than that of wind or solar power, enabling more energy production per unit area. In addition, wave energy exhibits a degree of predictability and stability not typically found in other renewable resources. Even after storms or subsidence, waves retain considerable energy-generating capacity, making them more reliable compared to intermittent resources^29^. For any wave energy converter, a fundamental consideration is the amount of power available in the wave. The wave power per unit crest length ($\:P$) can be expressed as a function of the wave’s significant height (*H*_*s*_) and peak period (*T*_*p*_). This provides the basis for estimating the extractable energy potential at a given site^30^. The wave power can be calculated using the following Eq. (1):

1 $$\:P=\frac{\rho\:{g}^{2}}{64\pi\:}{H}_{m0}^{2}{T}_{e}$$

Where $\:P$ is wave power per unit crest length (W/m), $\:\rho\:$ is water density (kg/m³), $\:g$ is gravitational acceleration (m/s²), *H*_*s*_ ​ is significant wave height (m), and *T*_*p*_ ​ is the peak wave period (s)^31,32^.

Physically, the significant wave height *H*_*s*_ and peak wave period *T*_*p*_ are the primary descriptors of sea-state energy and jointly determine the available wave-power flux incident on the coast. Water density and gravitational acceleration provide the fundamental scaling of this energy in the deep-water approximation. For the blowhole system, the outlet air velocity and outlet cross-sectional area govern the volumetric and mass flow rates of air, which directly control the kinetic energy available for conversion. Blowhole geometry, including conduit length and outlet diameter, influences pneumatic compression and discharge behaviour, thereby affecting airflow amplitude and intermittency^19,20^.

### Experimental model

**Fig. 4:**
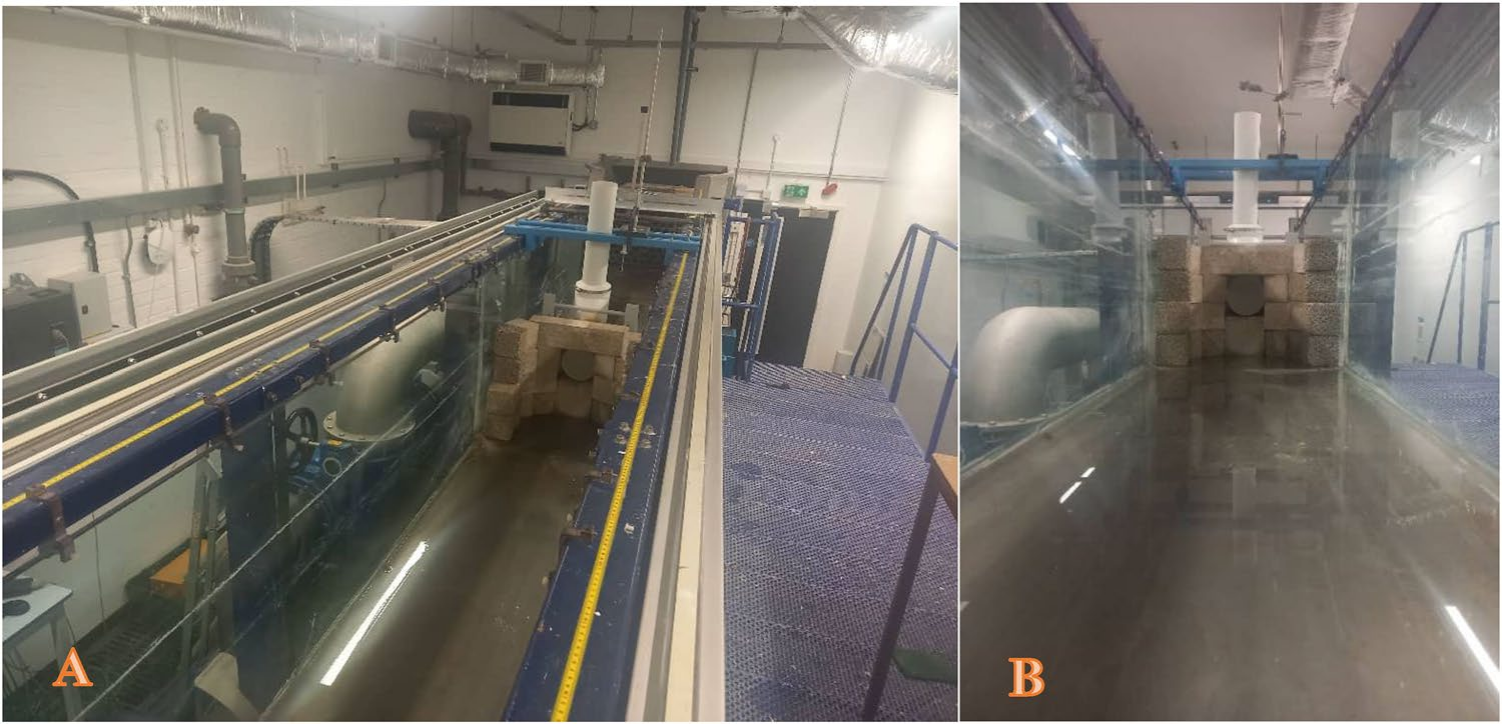
Laboratory flume model used to reproduce blowhole dynamics. (A) External view of the 5 m × 0.6 m × 1.2 m wave flume. (B) Internal view showing the arrangement of concrete blocks and the assembled blowhole conduit (horizontal pipe, elbow transition, and vertical riser). Scale bar = 0.5 m.

In this study, for the first time, the natural structure of the blowhole cavity is used as an innovative approach to generate energy from waves. The oscillating water column concept is used for generating energy. To evaluate the performance of a blowhole in power generation, some laboratory-scale physical model tests were carried out in a flume at the Engineering Department of the University of Exeter. The flume used to simulate the natural blowhole has a width of 0.6 m, a height of 1.20 m, and a length of 5 m (Fig. 4).

**Fig. 5:**
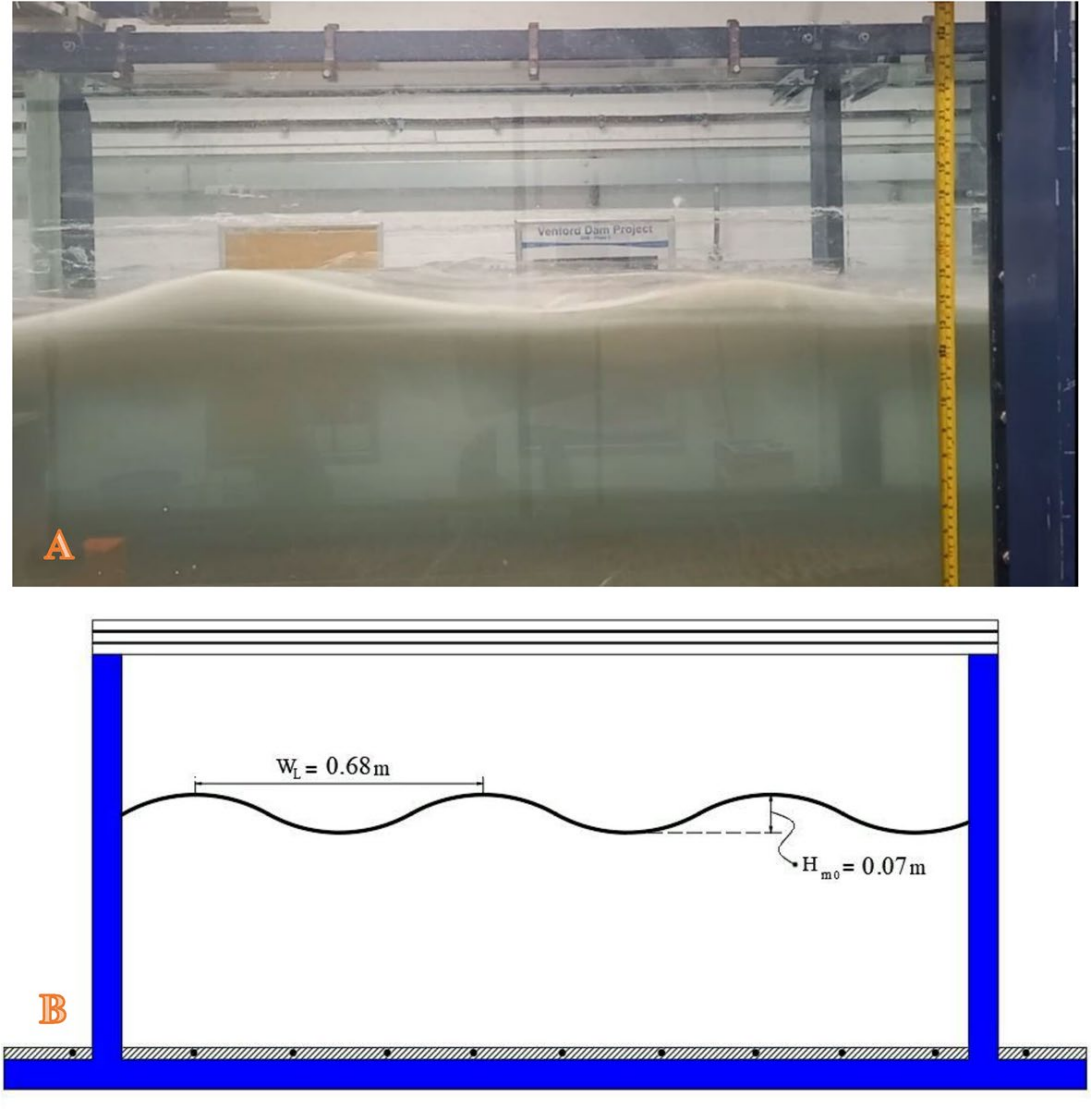
Wave conditions generated in the laboratory flume for model validation. The sample profile shows incident wave height of 0.07 m and period of 0.6 s under controlled forcing. Scale bar = 0.2 m.

The waves used in this experiment were created by a wave maker. In this experiment, we tried to produce waves with the same frequency so that the extracted results were repeatable. The generated waves were recorded by the camera and their height and return period were determined. Using a wave generator, the test wave was produced under controlled laboratory conditions, a sample of which is shown in Fig. 5. Concrete blocks in the flume were used to simulate the natural environment and a pipe with a bend at the bottom end was used to simulate the geometry of the blow hole. The pipe used to simulate the blow hole consisted of 3 parts: a horizontal part, an elbow, and a vertical part. The horizontal part was a 6-inch diameter pipe parallel to the axis of water flow and wave motion, which was placed underwater and placed between concrete blocks. To change the direction of the water flow and move it upwards, two 45-degree arcs of 6 inches were used to convert 6 to 4 inches. The vertical part was a tube with a diameter of 4 inches and a height of one meter, on which an anemometer was installed. A wind speedometer was used to measure the wind speed at the output point of the converter. However, the key factor in this part is the amount of system draft, which plays the role of model draft in the laboratory model of the speed measuring device. In the field-scale numerical simulations, established modelling approaches previously reported in the literature were adopted to account for effective draft representation^33,34^.

On the other hand, one of the key factors in the laboratory part of this project is the calculation of mass flow rate. Mass flow rate is one of the key factors because it serves as a primary criterion for comparing laboratory results with numerical calculations, especially in the validation of numerical models. Accurate determination of mass flow rate improves reliability and accuracy of experimental data and facilitates comparison and analysis of results. To calculate this value in laboratory results, the following Eq. (2) can be used^34–36^:

2 $$\:\dot{m}=\rho\:.A.v$$

where ρ is the density of the air (kg/m³), $\:A$ is the cross-sectional area of the pipe (m²), and $\:\nu\:$ is the velocity of the air at the outlet (m/s).

Also, by accurately measuring these parameters in the laboratory, an accurate mass flow rate is obtained, which not only supports numerical models, but also helps to identify any discrepancies between theoretical predictions and experimental results.

### CFD model

The main part of this research is to present a Computational Fluid Dynamics (CFD) model of the developed natural blowhole system. In the first step, laboratory conditions were simulated using ANSYS Fluent (commercial 2023) software. The CFD simulations were conducted using a two-phase Volume of Fluid (VOF) approach to capture the air-water interface and transient pressure pulses driving blowhole jets^37,38^. A Reynolds-Averaged Navier-Stokes (RANS) framework with the realizable k-ε turbulence model was employed to represent turbulent mixing within the cavity^39^. The computational domain replicated the laboratory geometry and was discretised using a hybrid mesh with local refinement around the blowhole throat to resolve high-gradient regions. A time step of 0.001 s ensured numerical stability and accurate tracking of the jet formation cycles. Grid-independence tests were performed by comparing jet velocity and mass-flow outputs across three mesh densities; deviations below 3% confirmed adequate resolution. Boundary conditions followed the laboratory forcing, with wave input at the flume inlet and atmospheric pressure at the blowhole outlet. The model was run for a duration sufficient to capture multiple oscillation cycles, and the resulting outlet velocities were extracted for comparison with experimental measurements. These are specifically designed for real-world application of the model, which in this case relates to the natural ocean environment. The main concept for this real-world model is outlined in Table 2.

**Table 2:**
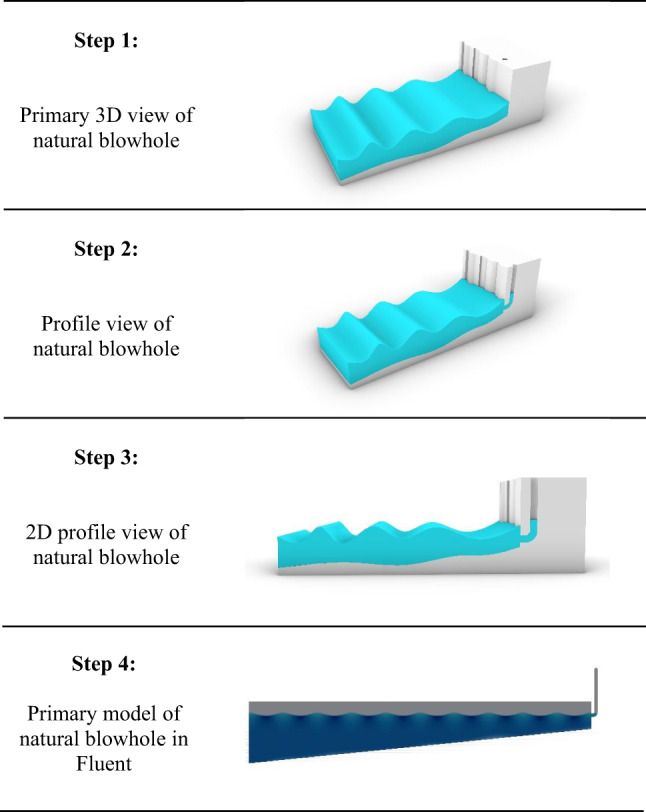
Conceptual modelling steps used to translate the natural Chabahar blowhole geometry into laboratory and CFD representations.

The CFD model is developed in a two-step process. First, the model is validated by a laboratory model. This validation includes identifying and applying appropriate boundary conditions. These boundary conditions are important because they simulate the real conditions encountered in the physical world, in this case, the ocean environment. The accuracy of these boundary conditions is important to ensure the reliability and accuracy of the model. In the second step, the general framework of the model is used for simulation in oceanic conditions. This step is important because it extends the applicability of the model from a controlled laboratory environment to complex and variable ocean conditions. A key aspect in determining these boundary conditions is the use of appropriate and correct theories. By validating the model in a controlled environment, it is possible to determine which boundary conditions most accurately describe fluid behaviour in real-world scenarios. This validation is essential in ensuring that the CFD model not only demonstrates theoretical principles but also closely matches the actual dynamics observed in natural environments, thereby increasing its accuracy and reliability^18,40^.

The last step in the simulation is to calculate the kinetic energy. Kinetic energy is one of the main factors that can be used to calculate the final output of models in an oscillating water column (OWC) system. Understanding the kinetic energy in the system allows accurate and efficient prediction, which can be critical in optimizing the design as well as performance of OWC devices.

The amount of kinetic energy required to run laboratory models requires two main variables: the mass flow rate and the air flow velocity at the outlet point. Mass flow rate determines how much mass of a fluid is moving through the system per unit of time, while air flow rate affects how fast the fluid moves. Together, these factors allow the calculation of kinetic energy, which is a measure of the energy available to a fluid^41–43^.

The wave conditions adopted in the experimental and numerical analyses are intended to represent characteristic nearshore sea states along semi-exposed rocky coastlines, where offshore wave spectra undergo transformation through shoaling, refraction, and partial breaking. Regular wave forcing is employed to enable controlled laboratory measurements and systematic CFD validation; however, these conditions are interpreted as idealized components of broader irregular wave climates. Field-scale relevance is addressed through the probabilistic Monte Carlo framework, which incorporates regional wave climate statistics and implicitly reflects variability in wave height and period. Although higher-order nonlinear wave interactions are not explicitly resolved within the CFD framework, their influence is partially captured through stochastic energy estimation and is acknowledged as an important extension for future work.

### Probabilistic framework (Monte Carlo)

The probabilistic analysis employed a Monte Carlo framework to propagate uncertainty in offshore wave conditions into blowhole energy estimates. Joint distributions of significant wave height (*H*_*s*_) and peak period (*T*_*p*_) were derived from the multi-year hindcast record and were fitted using non-parametric kernel density estimation to preserve the observed seasonal variability. For each of the 5,000 Monte-Carlo realisations, a pair of (*H*_*s*_, *T*_*p*_) values was randomly sampled from the joint distribution, and these inputs were converted into corresponding cavity pressures and outlet velocities using the CFD-validated response model. Each simulation produced a realisation of mass-flow rate and kinetic-energy flux, enabling the construction of full probability distributions and seasonal subsets. The resulting ensemble characterises both central tendencies and confidence intervals of blowhole performance under realistic ocean variability^32,44,45^. The framework was parameterized using wave climate data for Chabahar, derived from previous hindcast and observational studies. These studies report a mean annual wave energy flux of approximately 5 kW/m, with marked seasonal variation peaking at approximately 15.6 kW/m in July and dropping to approximately 4 kW/m in winter. Two principal sea-state classes were identified (Table 3):

**Table 3:** Principal offshore sea-state classes and occurrence frequencies used in the Monte Carlo framework.

Class	H_s_	T_*p*_	Annual frequency
Class 1	≈ 0.95–1.0 m	≈ 8–8.5 s	accounting for ~ 15% of the year
Class 2	≈ 1–2 m	≈ 10s	accounting for ~ 10% of the year

Lower-energy Sea states (*H*_*s*_ < 1.0 m) were also included to represent the remaining annual occurrence.

To account for measurement uncertainty, an error margin of ± 0.2 m was applied to (*H*_*s*_) and 5–10% variation to (*T*_*p*_), consistent with buoy validation studies. For each sample, the wave power per unit crest length was calculated using the equation already presented in Sect. 5.1. The resulting values were then combined with the validated relationships between air velocity, mass flow rate, and kinetic energy derived from the experimental and CFD models.

By repeating this procedure across thousands of simulations, the Monte Carlo analysis produced a probability distribution of blowhole energy output. This allows for the reporting of mean values, confidence intervals, and extreme scenarios, providing a more realistic and robust assessment of blowhole energy potential compared with single deterministic estimates. The number of Monte Carlo realizations determines the statistical stability of estimated wave-power distributions. Increasing the sample size reduces sampling error^46^ in the mean and improves representation of the distribution tails^47^, which are particularly important for assessing extreme but infrequent energy conditions relevant to structural loading and reliability. Monte Carlo simulation is appropriate here because the objective is to characterize the distribution of wave-power availability under uncertain sea-state forcing rather than resolve transient hydrodynamics for each realization. Sensitivity analysis indicates that key statistics, including the mean and 95% confidence bounds, converge as the number of realizations increases, with negligible changes observed beyond approximately 5,000 simulations.

## Results

### Experimental results

The laboratory flume experiments reproduced the characteristic pressure oscillations and air-jet formation observed in natural blowholes. Across all runs, outlet air velocities ranged from 4.1 to 7.6 m s⁻¹, with corresponding mass-flow rates between 0.12 and 0.27 kg s⁻¹. These values were consistent across repeated trials, with variability below 5%, indicating stable and repeatable behaviour. The temporal evolution of jet velocity exhibited a quasi-periodic structure linked to the incident wave period, and the peak values corresponded to the crest-driven compression phase within the cavity. These measurements provided the empirical basis for calibrating the CFD model and validating its predictive skill prior to field-scale extrapolation.

**Fig. 6:**
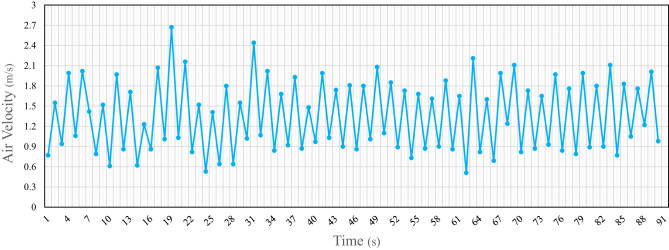
Time series of measured air-jet outlet velocity from the laboratory blowhole model over a 90 s test interval.

Also, in Fig. 6, the air flow velocity graph at the outlet point is measured at a specific time of 90 s for the laboratory model.

### CFD results

#### Validation

**Fig. 7:**
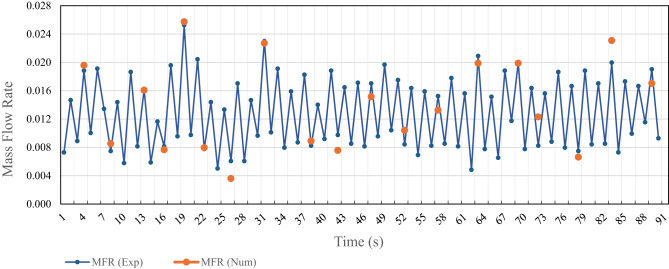
Comparison between experimentally measured and CFD-predicted air mass-flow rates at the blowhole outlet, used for numerical model validation.

**Fig. 8:**
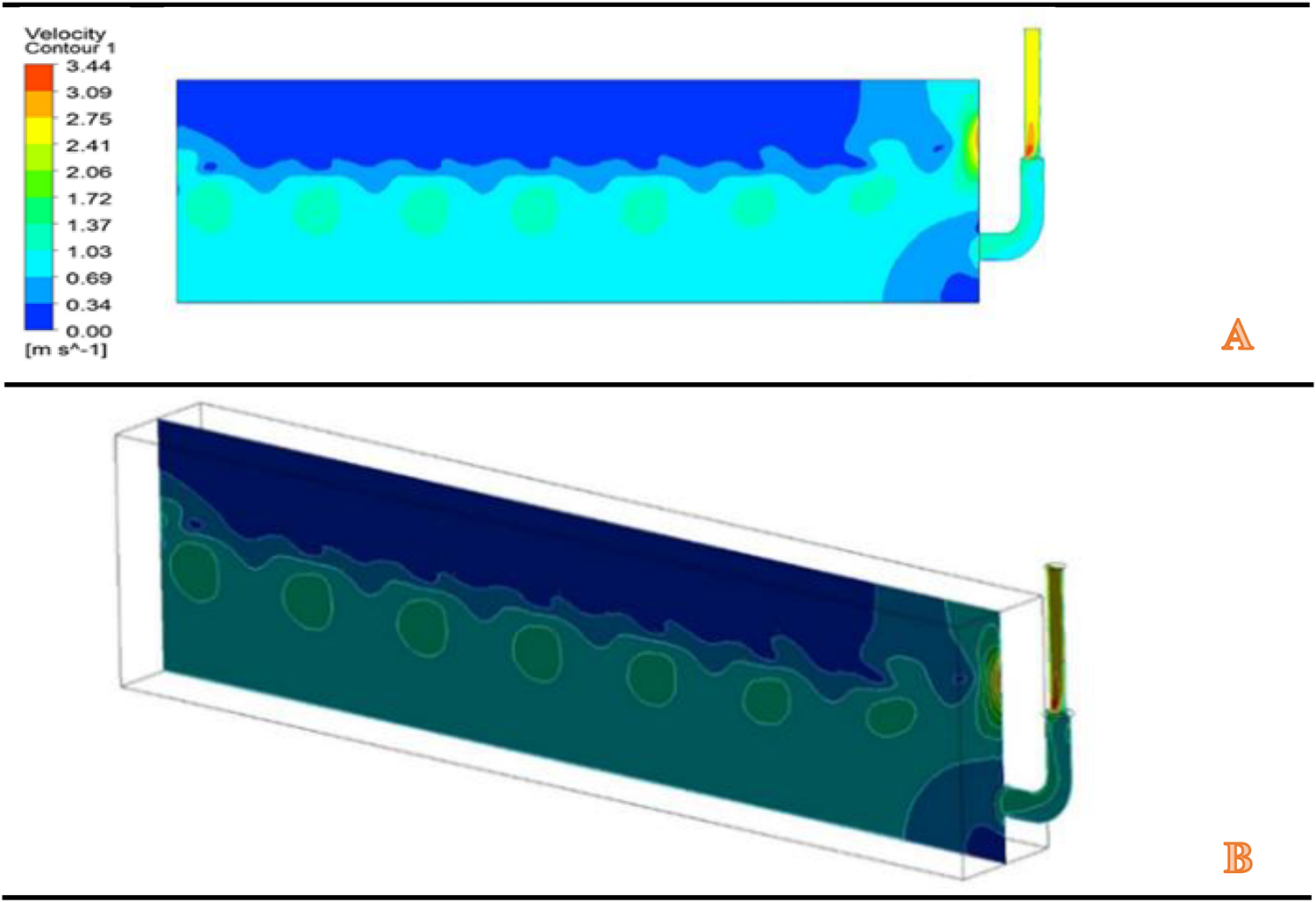
CFD validation of the laboratory blowhole model showing (A) two-dimensional velocity-field distribution at peak jet formation and (B) three-dimensional airflow structures within the cavity and outlet.

The data derived from simulations were compared against the results from laboratory experiments. According to Fig. 7, the calculation of the mass flow rate in the numerical model (MFR Num) corresponds with the mass flow rate of the experimental model (MFR Exp) with significant accuracy. This indicates the correct implementation of the boundary condition in the software model, which can enable simulations performed for real models with high reliability. For example, in Fig. 8 illustrates that the peak velocity at the blowhole outlet was recorded at 2.67 m/s, closely matched by the numerical simulations indicating a maximum velocity in the outlet of approximately 2.72 m/s (Fig. 8). This close agreement between the numerical and experimental data proves the simulations’ validity, demonstrating their reliability for the studied phenomenon. To quantify model accuracy, the Root Mean Square Error (RMSE) between experimental and CFD outlet velocities was 0.05 m/s, corresponding to a relative error below 2%. Similarly, mass flow rate deviations were within 3% across all tested conditions, confirming robust agreement.

In the simulation performed in the software environment for the desired blowhole, an effort was made to combine all the necessary factors to achieve accurate results. This involved using a realistic model that accurately represented the actual dimensions of the case study (Chabahar natural blowhole). The actual model shown under the existing boundary conditions and parameters that were validated by the laboratory model was implemented in the Fluent software environment.

**Fig. 9:**
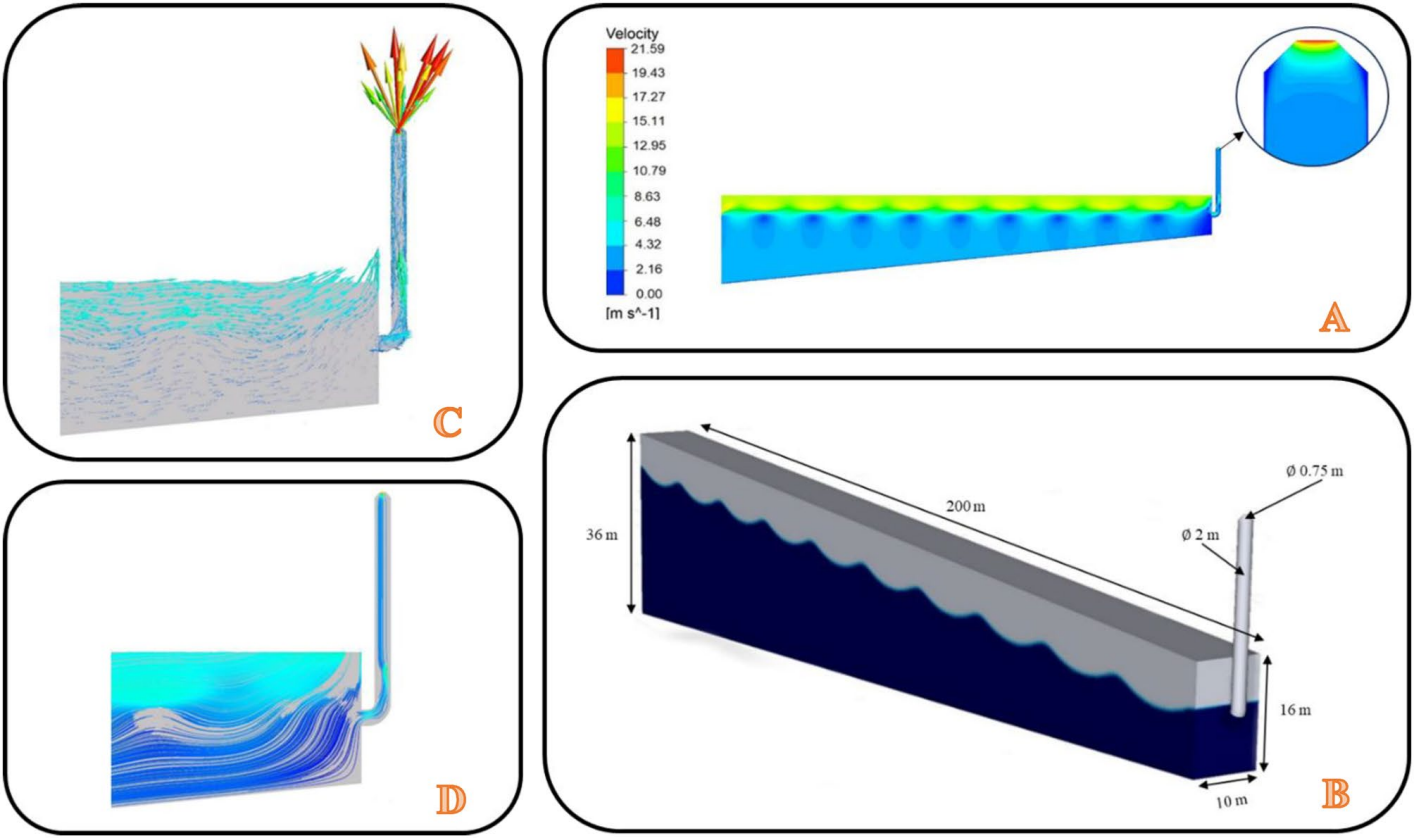
CFD simulation results for the full-scale Chabahar natural blowhole: (**A**) outlet velocity contours, (**B**) field geometry and dimensions used in the computational model, (**C**) velocity-vector field illustrating airflow acceleration toward the outlet, and (**D**) streamlines showing cavity oscillation and jet formation.

The data obtained from the CFD model output provides a comprehensive understanding of the power generation capabilities of a blowhole, which is clearly shown in Fig. 9. Here, the peak velocity at the exit point of the blowhole is 21.59 (m/s). Furthermore, considering the mass flow rate which is equal to 13.02 (kg/s), it is deduced that a significant amount of kinetic energy, approximately 3 (kW) can be harnessed from this natural phenomenon.

### Probabilistic (Monte Carlo) results

**Fig. 10:**
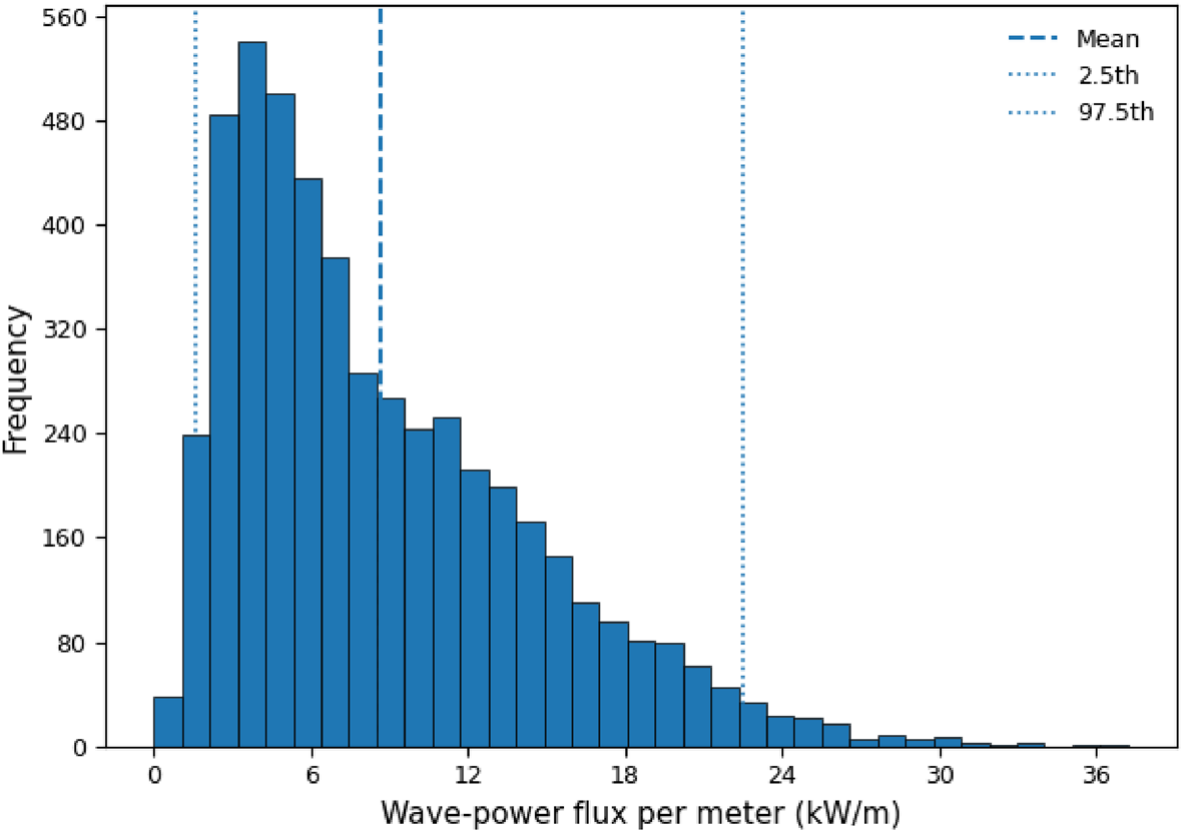
Histogram of Monte Carlo-derived wave-power flux per unit crest length based on 5,000 simulations, showing the distribution of energy availability under variable sea-state conditions.

The Monte Carlo analysis was conducted with 5,000 simulations, each generated by random sampling of significant wave height (*H*_*s*_) and peak period (*T*_*p*_) within the prescribed seasonal probability distributions. These inputs were propagated through the standard deep-water wave-power formulation and the validated experimental-CFD relationships to obtain a comprehensive set of energy flux values. Across all simulation, the present-day wave-power flux per meter exhibited a broad distribution (Fig. 10). The mean flux was 8.7 kW m^− 1^, while the median flux was 7.1 kW m^− 1^. The standard deviation of the distribution was 6.7 kW m^− 1^, reflecting the spread of simulated outcomes. The minimum value recorded in the simulations was 0.01 kW m^− 1^, and the maximum value reached 37.2 kW m^− 1^. The 95% confidence interval (2.5th −97.5th percentile) spanned from 1.6 kW m^− 1^ to 22.5 kW m^− 1^, indicating that many simulations fell within this range.

**Fig. 11:**
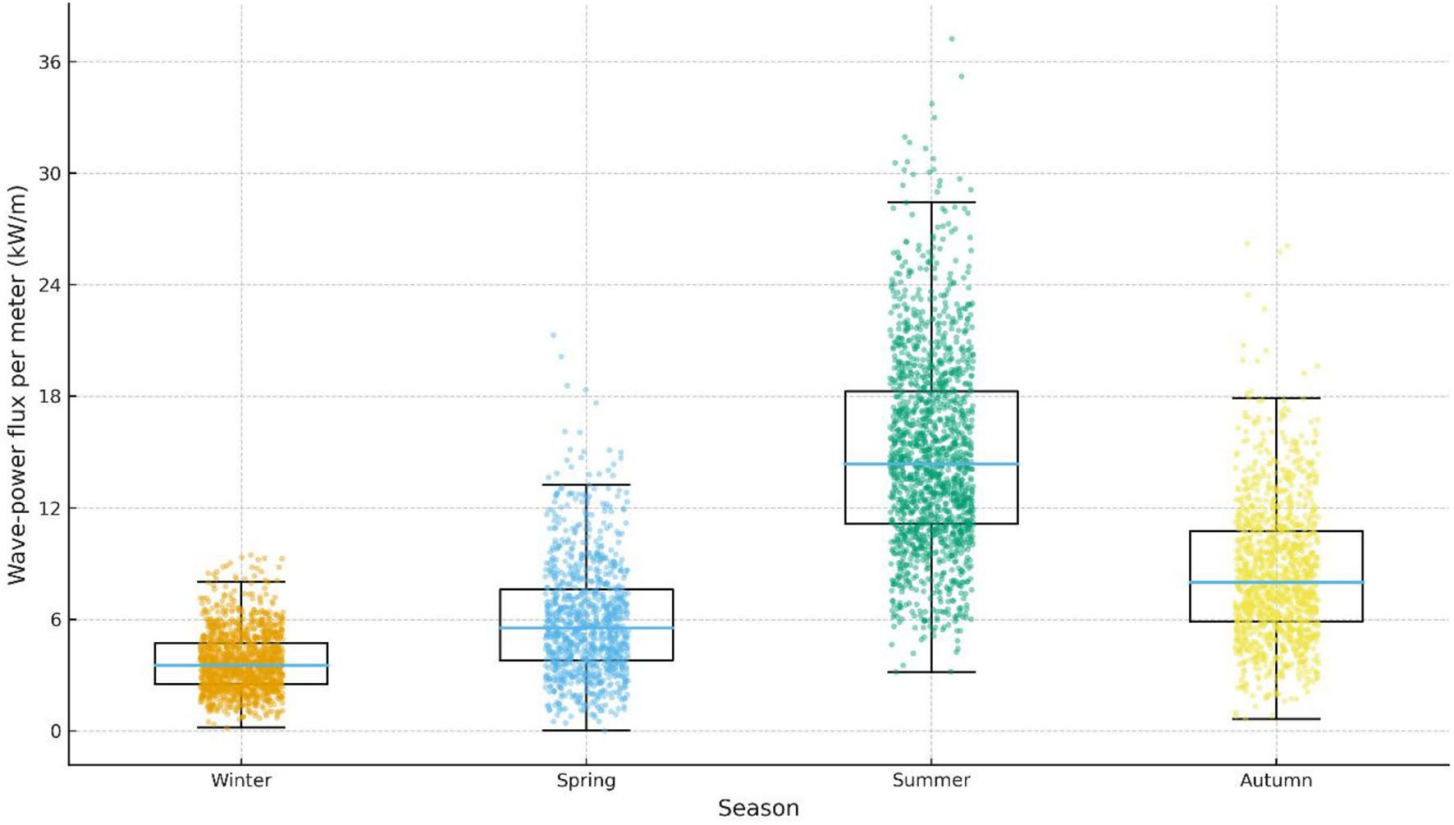
Seasonal distributions of Monte Carlo-simulated wave-power flux, highlighting differences in central tendency and variability between winter, spring, summer, and autumn.

When disaggregated by season, distinct statistical profiles were obtained for winter, spring, summer, and autumn (Fig. 11).

**Fig. 12:**
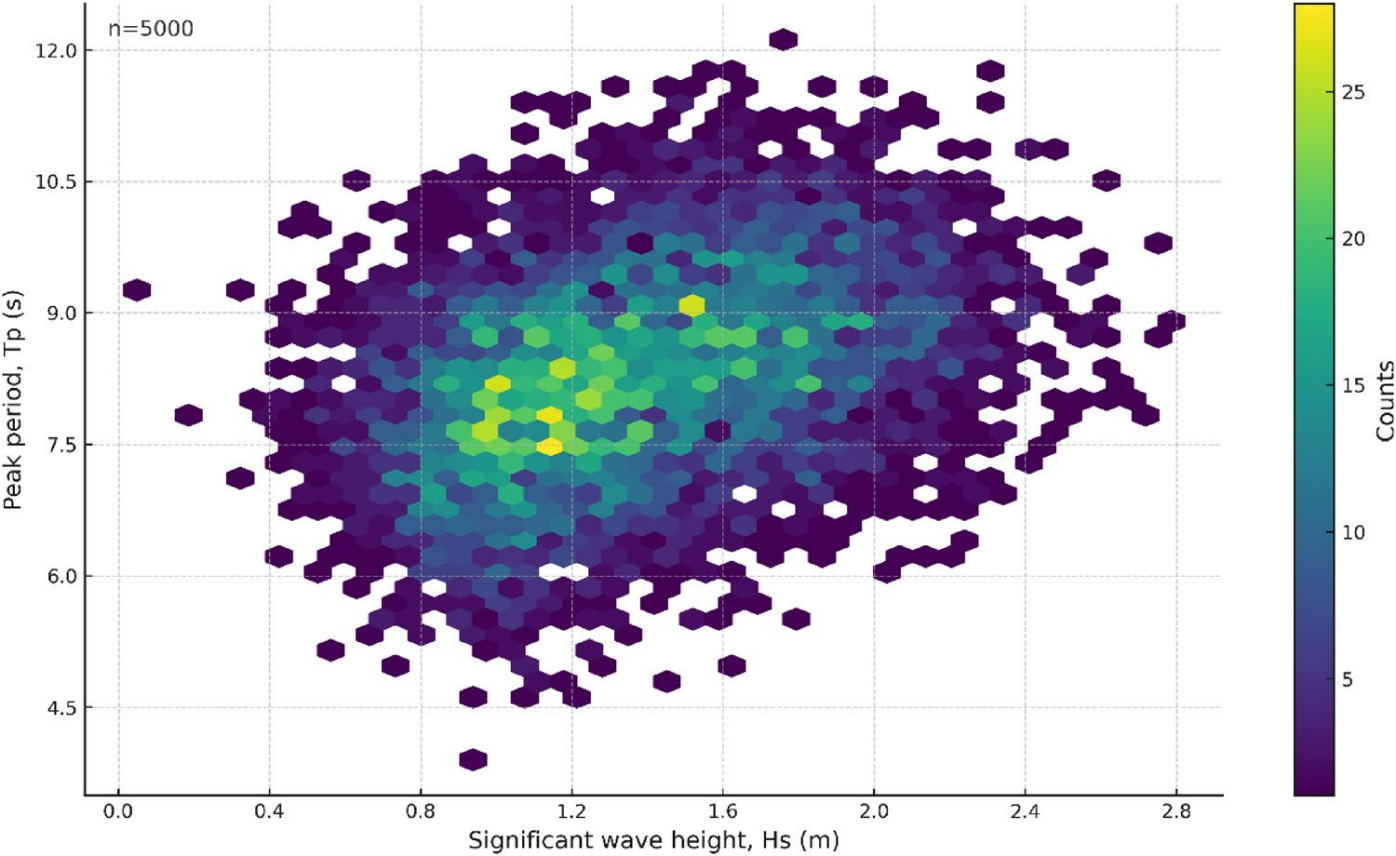
Joint probability density of sampled sea states used in the Monte Carlo analysis, showing the distribution of significant wave height (*H*_*s*_) and peak period (*T*_*p*_).

Seasonal sample sizes were between approximately 1,000 and 1,500 simulations per category, allowing stable estimates of central tendency and variability. Table 4 reports the mean, median, standard deviation, minimum, maximum, and confidence interval bounds for each season. The distribution of sampled sea states used in the simulations is illustrated in Fig. 12, showing the joint density of *H*_*s*_ ​and *T*_*p*_ ​. Most simulation were concentrated within the range of 0.5–2.0 m for *H*_*s*_​ and 6–10 s for *T*_*p*_, with the highest density near *H*_*s*_ ​≈ 1.2 m and *T*_*p*_ ​≈ 8s.

**Table 4:** Seasonal statistical characteristics of Monte Carlo-simulated wave-power flux per unit crest length (kW m⁻¹).

Season	*N*	Mean	Median	Std	Min	2.5%	97.5%	Max
Winter	1242	4.5	4.2	3.1	0.03	0.9	10.6	18.9
Spring	1008	7.0	6.6	4.4	0.01	1.3	15.2	25.5
Summer	1511	13.2	12.7	6.7	0.12	3.1	28.8	37.2
Autumn	1239	8.3	7.8	5.0	0.07	1.7	17.9	26.6

Seasonal variability has direct implications for long-term reliability and feasibility^48,49^. Higher summer wave-power flux increases the probability of sustained operation and coincides with elevated regional energy demand, whereas reduced winter flux implies periods of limited output. Seasonal probabilistic metrics therefore provide a more informative basis for planning than annual mean values alone.

The kinetic energy values reported in this study represent the mechanical energy associated with high-velocity air jets expelled through the blowhole vent and are intended as upper-bound indicators of extractable energy rather than direct electrical output. In practical applications, this energy could be coupled with a range of power take-off mechanisms employed in modern OWC systems, including bidirectional air turbines, impulse turbines, or hybrid pneumatic–electromechanical converters. Alternative concepts, such as piezoelectric or flow-induced vibration harvesters, may also be considered for low-power, distributed energy extraction in environmentally sensitive coastal settings. The present framework is therefore adaptable to multiple PTO configurations and serves as a basis for future techno-economic optimization.

## Discussion

The integration of experimental, numerical, and probabilistic approaches provides a holistic assessment of blowholes as a renewable energy system. Laboratory experiments confirmed that airflow generated by wave forcing can achieve velocities and mass flow rates sufficient to drive small turbines. CFD simulations reproduced these dynamics with high fidelity, enabling confident extrapolation to real-world geometries. The estimated kinetic energy output of ~ 3 kW for the Chabahar blowhole aligns with theoretical values for small oscillating water column (OWC) devices, reinforcing the feasibility of this natural analogue.

A key contribution of this study is the probabilistic framework, which moves beyond deterministic assessments. The Monte Carlo analysis revealed not only central tendencies (mean flux of 8.7 kW m⁻¹) but also the probability of extreme events, with outputs exceeding 30 kW m⁻¹ under favourable sea states. Seasonal contrasts were particularly strong: summer conditions dominated energy availability, while winter offered the lowest returns. This aligns with regional monsoon-wind forcing in the Oman Sea and demonstrates the importance of probabilistic modelling for energy reliability assessment.

The quantified airflow dynamics and probabilistic forcing characteristics have direct implications for future design of blowhole-based nature-based energy systems. The high peak outlet velocities inferred from field-scale CFD results indicate that power-take-off performance will be governed primarily by vent-level flow conditioning and turbine selection rather than by large structural modifications. Bidirectional oscillatory airflow necessitates the use of self-rectifying turbines or flow-rectification strategies, while nozzle or diffuser shaping may reduce losses and improve operational stability^50^. Importantly, the probabilistic energy distributions enable reliability-based sizing of power-take-off components by identifying exceedance probabilities for minimum operating thresholds and extreme loading events.

OWC-related studies generally report performance under prescribed or idealized wave conditions and focus on hydrodynamic or pneumatic efficiency of engineered systems. In contrast, the present study treats the blowhole as a nature-based energy asset and evaluates its performance through a validated experimental-CFD pathway coupled with probabilistic forcing. This distinction allows assessment of realistic energy potential and reliability for a fixed natural structure under uncertain and seasonally varying ocean conditions. Compared with engineered OWC devices, natural blowhole-based systems do not require large, constructed chambers or offshore installations, potentially reducing visual impact, seabed disturbance, and additional coastal infrastructure. The principal advantage of the NBS-OWC concept lies in leveraging existing geomorphological features rather than claiming superior energetic efficiency, thereby emphasizing minimal intervention and integration with natural coastal processes^18,20,51^.

Engineered oscillating water column devices are characterized by deliberately designed chamber geometries, controllable resonance characteristics, and standardized power take-off systems that enable systematic hydrodynamic optimization. In contrast, natural coastal blowholes constitute irregular, site-specific conduits formed through long-term geological erosion, with flow behaviour governed by local rock morphology, fracture networks, and nearshore wave transformation processes. From a scalability perspective, engineered OWCs are modular and replicable across sites with similar wave climates, whereas natural blowholes are inherently spatially constrained and cannot be reproduced without substantial geological modification. Consequently, performance metrics in the present study are expressed in terms of localized kinetic energy potential rather than device-level electrical efficiency, emphasizing feasibility and upper-bound energy estimates rather than direct equivalence with commercial OWC technologies^52,53^.

From an environmental and socio-economic perspective, blowholes offer distinct advantages over conventional offshore wave farms. Their integration into existing coastal landscapes minimizes additional infrastructure and reduces ecological footprints. They also avoid some of the visual, spatial, and ecological conflicts associated with large offshore arrays.

The quantitative results presented for Chabahar Bay are inherently site-specific. However, the methodological framework is transferable. Application to other regions requires re-parameterization of blowhole geometry, local wave climate, and boundary conditions, after which the same experimental-numerical-probabilistic workflow can be applied.

## Conclusion

This study provides the first probabilistic evaluation of natural blowholes as a viable wave-energy resource, integrating laboratory experimentation, CFD modelling, and Monte-Carlo analysis within a Chabahar Bay case study. The flume experiments successfully reproduced the characteristic dynamics of blowhole jets, confirming that the resulting outlet velocities and mass-flow rates are sufficient to drive turbine systems under controlled conditions. The numerical simulations closely mirrored the laboratory behaviour and enabled a reliable translation of these dynamics to field scale, yielding an estimated kinetic energy output on the order of 3 kW. By extending the analysis to 5,000 Monte-Carlo realisations, the study quantified the full range of uncertainty, producing a mean energy flux of 8.7 kW m⁻¹ with a 95% confidence interval spanning 1.6–22.5 kW m⁻¹. These results reveal marked seasonal contrasts, with summer conditions delivering the highest median flux (12.7 kW m⁻¹) and winter the lowest (4.2 kW m⁻¹), driven primarily by monsoon-modulated wave regimes. Taken together, the findings demonstrate that natural blowholes constitute a viable, low-impact and inherently place-based form of wave-energy extraction, offering ecological and social advantages over conventional offshore developments while expanding the portfolio of nature-integrated marine renewables. Natural coastal blowholes occupy a conceptual intersection between engineered oscillating water column technologies and emerging nature-based solutions for coastal renewable energy. Although they are unlikely to replace large-scale commercial wave energy converters, they offer a low-impact, site-adapted option for localized energy harvesting, environmental monitoring, or hybrid coastal infrastructure applications. Within the broader evolution of wave energy systems, blowhole-driven concepts illustrate the potential of leveraging existing geomorphological features to reduce construction impacts while enhancing coastal resilience. As future coastal renewable-energy strategies increasingly emphasize sustainability, adaptability, and ecosystem compatibility, natural blowholes may represent niche yet valuable components of diversified coastal energy portfolios.

### Applications, limitations, and future scope

The energy scale identified in this study suggests that natural blowholes are most suitable for small-scale and site-specific applications rather than grid-scale electricity production. Potential applications include coastal micro-generation for remote loads, power supply for monitoring and sensing infrastructure, navigation and safety lighting, and integration into coastal microgrids where seasonal energy availability aligns with local demand patterns.

Several limitations constrain the broader deployment of blowhole-based energy systems. Blowholes are inherently site-specific geomorphological features and cannot be replicated artificially without significant intervention. Their long-term structural stability may be affected by cliff erosion, conduit collapse, sediment infill, and extreme storm events, with additional uncertainty arising from sea-level rise and future changes in wave climate. Environmental and regulatory considerations may further restrict engineering intervention in locations where blowholes are ecologically sensitive or protected as natural heritage features.

Future research should focus on long-term field monitoring of pressure and airflow dynamics to validate modelled variability, integration and testing of power-take-off systems under oscillatory flow conditions, and techno-economic assessment incorporating reliability metrics derived from probabilistic and seasonal energy distributions. These steps are essential for translating conceptual assessment into practical deployment.

### Final synthesis

By positioning natural blowholes within the framework of nature-based solutions, this study introduces a novel pathway for sustainable coastal energy generation. While currently site-limited, blowholes represent an innovative complement to conventional marine renewables, bridging geoscience and engineering to support the global transition to low-carbon energy systems.

## Supplementary Information

Below is the link to the electronic supplementary material.

Supplementary material 1 (DOCX 2886.5 kb)

Supplementary Material 2

## Data Availability

The datasets generated and/or analysed during the current study are not publicly available because they form part of the author’s doctoral research materials, which remain under institutional restriction following the thesis defence and require formal approval from the supervising committee and the university’s research office for any external release. However, the data are available from the corresponding author on reasonable request, subject to obtaining the required institutional permissions.
